# Value of interventional radiology and their contributions to modern medical systems

**DOI:** 10.3389/fradi.2024.1403761

**Published:** 2024-07-17

**Authors:** Warren A. Campbell, Jeffrey F. B. Chick, David S. Shin, Mina S. Makary

**Affiliations:** ^1^ Division of Vascular and Interventional Radiology, Department of Radiology, University of Virginia, Charlottesville, VA, United States; ^2^ Division of Vascular and Interventional Radiology, Department of Radiology, University of Washington, Seattle, WA, United States; ^3^ Division of Vascular and Interventional Radiology, Department of Radiology, University of Southern California, Los Angeles, CA, United States; ^4^ Division of Vascular and Interventional Radiology, Department of Radiology, The Ohio State University Wexner Medical Center, Columbus, OH, United States

**Keywords:** interventional radiology, IR, value, scope of practice review, successful practices, comparative analysis, practice growth, IR payment models

## Abstract

Interventional radiology (IR) is a unique specialty that incorporates a diverse set of skills ranging from imaging, procedures, consultation, and patient management. Understanding how IR generates value to the healthcare system is important to review from various perspectives. IR specialists need to understand how to meet demands from various stakeholders to expand their practice improving patient care. Thus, this review discusses the domains of value contributed to medical systems and outlines the parameters of success. IR benefits five distinct parties: patients, practitioners, payers, employers, and innovators. Value to patients and providers is delivered through a wide set of diagnostic and therapeutic interventions. Payers and hospital systems financially benefit from the reduced cost in medical management secondary to fast patient recovery, outpatient procedures, fewer complications, and the prestige of offering diverse expertise for complex patients. Lastly, IR is a field of rapid innovation implementing new procedural technology and techniques. Overall, IR must actively advocate for further growth and influence in the medical field as their value continues to expand in multiple domains. Despite being a nascent specialty, IR has become indispensable to modern medical practice.

## Main points

• Interventional Radiology is a rapidly evolving specialty that provides value to all healthcare stakeholders through their collaboration, innovation, and dedication to patients.
• IR physicians are uniquely trained to provide minimally invasive procedures that offer safer, faster, and more cost-effective options for patients.
• With the evolution of value-based reimbursement models, IR physicians can adopt clinical patient models to supplement their reimbursement and patient volume.
• IR needs to practice multisystem marketing and advocacy to grow their practice, broaden their public influence, and recruit new talent to the specialty to expand their international impact.

## Introduction

Since the inception of interventional radiology (IR) by Charles Dotter in 1963, IR has proven to be an integral part of patient care. Advancements in imaging technology have broadened the scope of visually guided minimally invasive procedures to improve patient outcomes (1). IR's guiding principle is to reduce trauma via minimally invasive access with the goal of reducing patient recovery time, postoperative complications, healthcare costs, and achieving greater clinical outcomes. IR optimizes the “triple aim” of modern healthcare: improving patient experience (quality & quantity), improving the health of populations, and reducing per-capita costs (2). In the delivery of complex medical care IR provides value at all levels of patient care and to the associated stakeholders in the healthcare system.

The practice of IR in medicine has expanded over the past. IR in oncology spans from detection, diagnosis, therapeutic delivery, and continued management (ports, drains, etc.). The value of the IR market is expected to exceed 43 billion by 2029 with an estimated growth rate of 7.13% (3). The IR ecosystem is expanding into mixed clinical care models. Physicians are integrating inpatient and outpatient care with both procedural and longitudinal care. The resulting increase of billing for evaluation and management (E&M) are showing increases of 722% and 669%, respectively (4, 5). IR continues to be one of the most innovative fields of interdisciplinary medicine that is positioned for significant growth, investment, and prominence in the healthcare industry.

IR has a responsibility to demonstrate value at all levels of medical care. As one of the more nascently recognized medical specialties in 1994, IR physicians have identified a growing need to prove its value to external stakeholders, including hospital executives, insurance providers, hybrid IR/DR practices, referring specialists, patients, and to the general public (6). A common critique among healthcare specialists is that IR physicians are “masters of none,” and face increased competition for patient flow among other interventionalists (neurosurgery, vascular, cardiology, pulmonary, etc.). The purpose of this article is to review the domains of IR value and highlight important components of IR's success. This work reviews key functions of the IR physician's practice, outlines the financial value to patients, the profitability within different payment models, infrastructure requirements for successful practice, and defines important initiatives for continued advancement of the field.

## Methods

This is a descriptive review on how IR provides value to the medical system. This paper will review the advantages of IR procedures to patient outcomes, their cost-benefit analysis, and how IR practice can succeed in payment models. We also review the necessary factors including infrastructure, facilities, patient networking, and physician recruitment and retainment strategies for IR to be competitive in healthcare. Lastly, this work discusses future efforts that must be taken to communicate this value to healthcare specialists and the public at large.

In the review of comparative studies highlighting the medical or financial benefit of IR specific procedures, only studies in the last 25 years were included for relevancy. The exception to this cutoff was if no more recent comparative study has been performed or identified on literature search for a specific procedure. Studies referencing mixed results of an IR procedure's value or efficacy to surgical or medical alternatives addressed.

## Outline

1. Scope of Practice
  a. Diagnostics
  b. Clinical & Consultation Services
  c. Minimally Invasive Procedures
2. Minimally Invasive IR Procedures
   a. Nonvascular Procedures
   b. Vascular procedures
3. Medical and Financial Value of Minimally Invasive Procedure
   a. Medical Benefits
   b. Financial Benefits
   c. Research & Innovation
4. Financial Contributions
   a. Direct Contributions
   b. Indirect Contributions
5. Revenue Analysis of IR Reimbursement Models
   a. Fee for Service
   b. Value Based Care
   c. Bundled Payment
   d. Capitation
6. Elements of Successful IR Practice
  a. Recruitment
  b. Marketing
  c. Networking
  d. Incentives
  e. Expectations
  f. Infrastructure needs
7. Expanding the Impact of IR
  a. Promotion and Awareness
  b. Governmental Policy and Healthcare Infrastructure
  c. Future Directions

## Scope of practice

IR services are diverse and provide interventions at all stages of disease. The role of the IR physician spans beyond the surgical suite, where hospital systems rely on their skills in image analysis, consultation services, and patient clinics.

### Diagnostic role in data acquisition and analysis

IR physicians assist in patient data collection. They acquire angiograms for venous and arterial pathology. Nonvascular studies include lymphangiography and arthrography. IR has become the preferred specialist for ultrasound (US), computed tomography (CT), or magnetic resonance (MR) guided biopsies and has significantly reduced the need for open surgical sampling without sacrificing sample quality. Minimally invasive venous sampling collected by IR physicians supplements imaging data for the localization of endocrine tumors, such as parathyroid, pituitary, adrenal glands, and islet cell tumors where imaging is indeterminate, or laterality is uncertain. IR provides valuable information to specialists who rely on high quality images and sampling data for their clinical management.

IR physicians serve the important concomitant responsibility interpreting noninvasive imaging studies. IR confidently interprets MR, percutaneous and endoscopic/intravascular US, CT, angiography, and fluoroscopy. Surgeons and oncologists rely on discussions with trained radiologists for complex patient care and procedural evaluation. This extends to outpatient care where specialists rely on image analysis for disease management including prevention, detection, diagnosis, therapy monitoring, and prognosis. The focus on these diagnostic duties is highly setting dependent. Academic center IR physicians focus more on procedural tasks, where private practice physicians focused more on diagnostic interpretation (7).

### Consultation services are essential for advanced disease

In oncology patients, providers are challenged to find a meaningful therapeutic strategy to improve quality of life with minimal risks of intervention. IR serves as a valuable member of the tumor boards to provide their expertise in the development of a care plan. Bringing IR into the conversation can provide additional expertise on when nonsurgical options become viable.

IR can offer unique procedural options for challenging clinical cases. As consultants, IR can address internal referrals for vascular and localized procedures for untrained clinicians or offer experienced advantages over obstetricians and surgeons that do not perform these procedures regularly in their practice. By collaboration on patient care, the referring physician can synergistically benefit patient expense, experience, and outcome.

### Patient clinics provide longitudinal patient care

There are many financial and intangible qualities to incorporating patient clinics into IR practice. These allow greater involvement in evaluation and management of their own patient population and greater control over their pre-procedural and post-procedural pipeline. Office visits and follow-up provide an important service in building patient rapport and establish an element of public relations that would otherwise be impossible from procedural referrals alone. This step complements the other functions of IR and elevates the importance of their skills to the public (8).

### Specialized minimally invasive alternative therapy

IR's suite of minimally invasive strategies can be applied to an array of anatomical structures to accomplish various goals including tissue drainage, revascularization, reconstruction, repair, reinforcement, embolization, obliteration, and more. These procedures can serve as first-line therapies depending on patient pathology. Importantly, these therapies are alternatives to the traditional surgical and medical treatments that have a unique safety and efficacy profile particularly in poor surgical candidates and critically ill patients.

## Minimally invasive IR procedures

**Figure 1:**
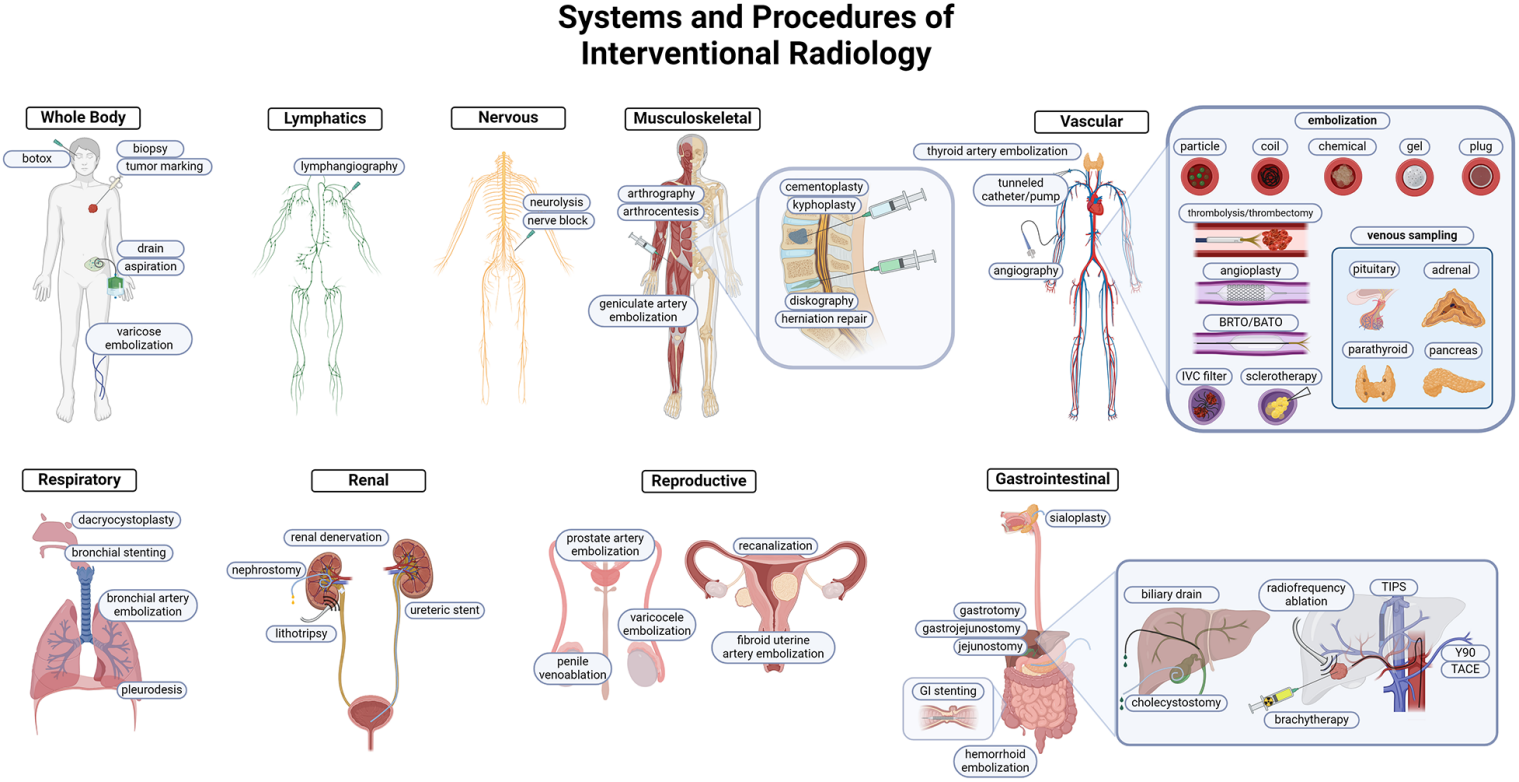
Overview of IR procedures. IR uses minimally invasive techniques to target pathology in every organ system. Each system highlights common and important interventions performed by IR. Targeting methodology commonly utilizes vascular access, and the prominent tools are highlighted in the cardiovascular system schematic. Both arterial and venous embolization, angioplasties, thrombectomy/thrombolysis, and venous sampling are integral to the IR toolkit.

The use of MR, US, CT, and fluoroscopy made visually guided procedures the forefront of innovation in patient care. The suite of tools and skills available to the IR physician made a wide variety of organ system interventions both safe, feasible, and practical. Some procedures offer a unique alternative to medical and surgical management for patients, while others are critical interventions that are instrumental to patient survival (Figure 1).

## General procedures

### Biopsies

IR is the most common operator of biopsy procedures because of their historical success in obtaining quality samples with less risk than open surgical retrieval. Biopsies performed using US, MR, CT, and positron-emission tomography (PET) are extensively documented and findings show high rates of success with few adverse events (9). Needle biopsies are safer, cheaper, and faster and can achieve sufficient samples in most clinical scenarios. While some scenarios benefit from open biopsies with higher rates of diagnostic success, there is often sufficient indication to trial needle biopsies first due to their high safety profile. In biopsy samples where the target is perivascular or difficult to access percutaneously, trans-vascular approaches can be taken to biopsy the liver and kidney (10, 11).

This function has become increasingly important in recent decades in oncological treatment. Initially the value was in cancer staging but has expanded to pathological analysis of molecular biomarker testing including gene expression, biomarker expression, cell signaling pathways, and other tumor indicators of tumor heterogeneity. IR serves as a critical intersection between the oncologist's medical management and the pathological characterization of the tumor (12).

### Image guided tumor marking

The precise marking of malignant lesions is a crucial step prior to surgical excision where margins can be challenging to delineate. Indication criteria have been generated based on the frequency of failed tumor localization or positive margins on resection (13). Wire and radioactive seed localization (RSA) are two techniques commonly used in breast cancer, with evidence showing RSA has improved outcomes for breast and lymph node biopsies (14–16). Other procedures where marking is indicated include pulmonary nodules, liver metastasis, hepatocellular carcinomas (HCC), and bone lesions. These IR procedures are valuable steps to minimizing follow-up surgeries and higher rates of complications.

### Image guided percutaneous drains and aspirations

With symptomatic abscess formation, image-guided percutaneous drainage can obviate the need for surgical intervention. IR physicians have become particularly useful in the treatment of pelvic, subphrenic, epigastric, urogenital, diverticular, appendiceal, and hepatobiliary abscesses (17). Antibiotic penetration into large confined thick-walled abscesses is poor, and IR is an indispensable resource for the management of localized infections. Their interventions significantly reduce morbidity and mortality of complex septic patients and provide source control.

### Hemostasis in trauma

IR has a growing role in providing hemostasis in trauma cases (18, 19). For acute hepatic laceration and hematomas, angioembolization is an approach to reduce the high rate of mortality. With excessive exsanguination, identifying the source of the bleed for operative repair can be impossible, allowing IR to both identify and embolize the source. With severe AAST (American Association for the Surgery of Trauma) liver grade IV or V injuries, IR intervention reduces mortality in conjunction with open surgery (20). In the setting of a hemodynamically stable patient with blunt or penetrating (e.g., gunshot wound) hepatic trauma, angioembolization can be sufficient for treatment for AAST II and III hepatic injuries. Based on this evidence, the Society of IR (SIR) has generated guidelines for the practicing physician to intervene in hepatic trauma (21).

Similarly for splenic lacerations, there is limited data to support either splenectomy or splenic artery embolization and often is dependent on center-to-center preferences. For Grade IV-V lacerations, embolization is often preferred with some evidence showing a decrease in infection risk without a change in mortality (22). Embolization can also be utilized pre-operatively for a planned splenectomy to improve operative time and reduce blood loss (23). However, the therapeutic appropriateness of embolization only applies for patients that are hemodynamically stable.

Pelvic trauma is another injury that commonly presents with organ compromise and associated pelvic vascular injury with high mortality rates. In cases with proven extravasation, selective and super-selective angiography can identify the origin of vascular compromise with simultaneously embolization. Thus, in many trauma centers the involvement of an IR team has become a vital component of their operation (24).

## Vascular interventions

IR physicians have demonstrated advanced skills for vascular access and intervention. The ability to obtain high quality angiography for diagnostic purposes, and simultaneously deliver therapeutics offers an efficient healthcare delivery model for patients. There are a wide set of diseases where vascular access is important for treatment. IR consults with oncologists, urologists, gynecologists, gastroenterologists, pulmonologists, hepatologists, and other vascular specialists to address vascular pathology in common diseases in their patient population. IR procedures are often first line therapy for management, and newer procedures are becoming increasingly advantageous to other alternative therapies and expanding their role in patient care.

### Central lines and ports

Hospital systems commonly utilize IR services for central venous catheters and tunneled lines/ports. Interventional radiologists can perform these procedures very quickly, cost-effectively, and with fewer complications than surgical placement. This contention is controversial. Given the direct methodology, the procedure can be performed successfully by other specialists and advanced practitioners. The procedure can be done safely in an outpatient setting as well. Many specialists would prefer to manage their patients internally, thus larger studies are required to better study the risks based on operator, setting, and equipment.

### Endovascular angioplasty and angiography

IR was the original pioneer of the experimental angioplasty to restore vessel integrity, and since that inception has become a cornerstone of vascular therapies (1). The practice of vascular procedures has shifted over time. With vascular IR growing in scope and capabilities to perform endovascular interventions, some were concerned that they would impede the practice of vascular surgery. Vascular IR physicians are frequently the source of diagnostic arterial and venous angiograms for evaluation of vascular disease. Neither practice was disrupted, as the treatment of peripheral arterial disease still largely lies with vascular surgery. According to Medicare data, IR performs only 25% of these procedures (25). However, IR's role in this space has been growing incrementally over the years in the US (26). In Europe, IR physicians are often the primary interventionalist since they often have the hybrid OR requirements for endovascular procedures (27).

This is not to say that endovascular procedures are not competitive among physician specialties. There are many cases where IR physicians were frontier proceduralists in difficult endovascular diseases including heart and brain catheterization. The primary issue here is that they controlled the patients and once they were trained in the technique it was incentivized to manage the patients internally. Neurosurgeons also argue that they are the most qualified to manage neurovascular procedures and their neurocritical care to have the lowest rates of major complications (28). In practice, there is collaboration between specialties in patient care and intervention may be dictated more by hospital resources and staff (29).

### Embolization

Embolization is the primary and essential tool in resolving many systemic diseases. The ability to percutaneously gain vascular access, and to achieve endovascular localization of pathology is essential to the benefits yielding IR. Significant research into embolic agents have provided more tools and options for IR to address unique pathology. Components range from coils, balloons, plugs, particles, liquids, and foams. The sub-specialization of equipment and combinatorial approach has refined the practice to confidently limit perfusion without causing ischemic damage to healthy tissue. IR can apply these agents to both arterial and venous systems as appropriate. While the success of these different agents can be operator dependent, they have exploded in popularity thanks to IR because of the safety profile and high efficacy rates (30).

### Transvenous obliteration

In systems of recurrent venous bleeding common in portal hypertension where esophageal and gastric varices are distended, venous obliteration by IR has been shown to produce the most reliable results until the liver disease can be addressed (31). Venous obliteration may also be more effective than TIPS, provided it's a more localized solution to destroy the venous system to prevent further bleeds (32). Further variants based on operator preference, speed, costs, and effectiveness can be selected such as using a plug (plug assisted retrograde transvenous obliteration, PARTO), coils with gel foam (coil-assisted retrograde transvenous obliteration, CARTO), or can be approached from systemic circulation for anterograde obliteration (balloon occluded antegrade transvenous obliteration, BATO). These customization options allow this procedure to have a high rate of success with few complications when sclerosing agent is successfully directed to the target varix.

### Balloon venoplasty and venograms

Because endovascular interventions to address venous obstruction have high rates of technical success, IR is offering first line approaches to resolving venous pathologies. Numerous etiologies cause venous fibrosis causing outflow obstruction such as central venous catheters, hemodialysis catheters, radiation exposure, trauma, or strictures. This may be symptomatic or cause interventional issues in gaining central venous access past the obstruction. Commonly IR will intervene in the subclavian, axillary, and brachiocephalic veins with balloon angioplasty or iliofemoral veins with balloon venoplasty with high rates of success (33). In the diagnostic evaluation of chronic venous disease, IR will obtain quality venograms.

Venograms are also important in diagnostic evaluation of portal hypertension to determine if the source of obstruction is prehepatic, intrahepatic, or posthepatic. Where Budd Chiari is identified, a combination of steps involving venoplasty, thrombus maceration, and stent placement is all within the procedural confines of venous reconstruction performed with IR (34). Successful drops in portal hypertension will prevent the patient from having surgical reconstruction and shunt placement.

### Endovascular aneurysm repair

Vascular rupture from aneurysms signifies a life-threatening emergency from large vessels in the thorax, abdomen, and cranium. Large aneurysms were traditionally addressed with open surgical repair. Large structural compromise of the aorta or common iliac vessels can be stabilized with the placement of a graft. Endovascular aneurysm repair (EVAR) now represents the mainstay of elective treatment (35, 36). Smaller vessels can be reinforced with coils or clips delivered through intravascularly. Large tube aortic stent grafts via a percutaneous femoral approach have been validated to be a safe and durable alternative to open surgery. However, there is still a lack of comparative studies to create confident guidelines when either open or endoscopic approaches would be appropriate in elective settings.

### Inferior vena cava (IVC) filter placement/retrieval

IVC filters commonly used for prior venous thromboembolism with contraindications for anticoagulation have become an IR dominant procedure. While they were traditionally placed by surgeons, advancements in minimally invasive fluoroscopy have made IR aptly trained for efficient and safe placement (37). Compared with surgeon or OR placement, IVC placement by IR has significantly reduced the cost, time, and complications of traditional surgical placement (38). Similarly, IR can apply similar techniques for the snare and retrieval of IVC filters for outpatient surgery.

## Respiratory

### Bronchial artery embolization

In the control of moderate to severe bronchial hemoptysis where medical management has failed, patients often need to resort to bronchoscopy or surgical treatment to provide hemostasis (39). Bronchial artery embolization has proven to be a safe and effective method to provide reliable hemostasis (40–44). While there is a high rebleed rate, the combination of more permanent embolic agents such as coils and polyvinyl alcohol or N-Butyl-2-Cyanoacrylate glue can ensure both immediate technical success and sustained hemostasis until the underlying pathology can be managed further. In emergent situations, IR is often the only effective bridge toward more definitive therapies.

### Bronchial biopsy and stent placement

Interventional pulmonologists have specialized in the use of bronchial scopes for the placement of airway stents to alleviate malignant obstruction (45). IR can fluoroscopically approach an endobronchial biopsy sample for masses proximal to the airway, and if there is sufficient access, it is logical that IR place the stent to maximize efficiency for the patient. This has been found to be safe and effective, demonstrating that IR can save patients from undergoing multiple separate procedures.

### Dacryocystoplasty

When the obstruction of the nasolacrimal duct causes epiphora, the definitive treatment is dacryocystorhinostomy. IR can offer an alternative dacryocystoplasty or stent placement that does not require general anesthesia to be performed (46). There are few absolute contraindications where neoplasm or dacryocystitis is observed, suggesting a broad population with epiphora could be offered this therapy as a first line therapy. While the long-term success rate is lower than surgical management, mild cases now have a safe and quick attempt to resolve the obstruction and associated symptoms.

## Musculoskeletal

### Arthrography

The injection of contrast into the joint space can provide enhanced CT or MR resolution for indeterminate joint disease. IR can inject contrast into the tibiofemoral, glenohumeral, ulnar radiocarpal, and tibiotalar joint spaces to provide increased sensitivity over CT or MR alone to correlate with physical exam finds (47–50). A positive finding using arthrography can sometimes prevent the more invasive arthroscopy. IR is particularly valued in pediatric arthrography with their advanced ultrasound navigation to guide contrast deposition.

### Provocative discography

The injection of contrast into the nucleus pulposus to correlate patient symptoms and disc disease has been controversial for its historically high false-positive rates and questionable clinical utility after significant advancements in MR imaging modalities (51, 52). There is also a risk of damaging the annulus and increasing the risk of future herniation (53). Despite this risk, MR findings have a false positives and spinal fusion or laminectomy with discectomy carries significant postoperative complications. With advancements in procedural techniques and tools to control the contrast manometry, IR's role in this complementary test can provide unique insight into the etiology of back pain in the right patient population (54).

### Vertebroplasty and kyphoplasty

The use of vertebroplasty for osteoporotic fractures was a common IR procedure used for pain and stabilization, but dropped in prevalence after the release of randomized controlled trials showing no statistical changes in these outcomes against sham placebo controls (55, 56). Further refinement in the procedure and inclusion criteria has allowed for continued use of the procedure and evidence of its effectiveness in reducing pain (57). This result is similar with kyphoplasty, where correction of distorted vertebral height relieve pain and limited mobility (58–60). Whether either procedure is superior is controversial and highly user dependent (61). IR should be consulted about spinal pain and the nature of their vertebral or disc pathology if they are a viable candidate because it can be performed quickly in an outpatient clinic to provide pain relief.

### Geniculate artery embolization

In the treatment of medication resistant osteoarthritis, patients with evidence of neovascularization and synovitis can embolize this vasculature to reduce inflammation and further degeneration (62). This is a novel approach receiving some preliminary validation in small cohort studies, that demonstrates technical success of reducing synovitis and improving functionality presumably from pain reduction. While the long-term success of this procedure is unstudied, this could serve as a robust complementary therapy with corticosteroids, hyaline injections, and physical therapy to preserve articular integrity and function prior to arthroplasty.

## Hepatobiliary

### Tunneled peritoneal catheters

Tunneled peritoneal catheters provide a dialysis option for patients with kidney failure (63). Surgeons and nephrologists can place these catheters, where IR is only utilized around 5% of the time (64). Percutaneous placement has shown to be safe and effective, and also found to be more cost effective than hemodialysis (65, 66). While a minority of IR physicians place these, having additional imaging and fluoroscopic equipment can provide additional confirmation about proper placement (67). This percutaneous peritoneal access can also be used for malignant ascites, where this approach was more cost effective after several paracentesis (68). This another procedural opportunity for IR to demonstrate proficiency in this technique to benefit patients of oncological and kidney diseases.

### Transjugular intrahepatic portosystemic shunt (TIPS)

When a patient with elevated portal pressures suffers from life-threatening recurrent variceal bleeding, TIPS is instrumental to minimizing future bleeds used in conjunction with beta-blockers, octreotide, vasopressin, and endoscopic ligation. TIPS can prevent bleeds longer than medications or endoscopic procedures alone due to the ability to equalize portal pressures (69–71). As a result, performed at early stages of cirrhotic disease is a cost saving measure that reduces the chances of a life-threatening bleed (69, 70, 72). While this does significantly increase the risk of hepatic encephalopathy, the clinical team balances the relative risks to mitigate immediate risks as the patient awaits transplant.

### Yttrium-90(Y90)/transarterial chemoembolization (TACE)/brachytherapy

Interventional oncology plays a significant role in the treatment and management of hepatobiliary tumors from hepatocellular carcinoma (HCC), cholangiocarcinoma, or metastasis (73–76). Working in conjunction with the surgical oncologist, minimally invasive procedures focus on curative ablation, reducing tumor burden, or inducing contralateral hypertrophic compensation for lobe resection. The choice in using Y90 or chemotherapy transporting beads to tumor sites is variable depending on the institution and the preferences of the oncologist and interventional oncologist, but evolving evidence suggests that radioembolization produces more robust patient outcomes (77). Similarly, the implantation of a radioactive I-125 seed can provide localized radioactive therapy with positive results when used in combination with other therapies.

### Tumor ablations

In the setting of hepatocellular carcinoma, minimally invasive techniques can be attempted as a curative therapy (78). Radiofrequency ablation targets a probe with low frequency currents that provide thermal stress to the tumor. Microwave ablations use higher frequency waves that generate heat stress inducing coagulative necrosis. Both techniques are frequently evolving and improving in their overall survival rates and data from several randomized trials are being collected. Current recommendations suggest only reserving this therapy for individuals who are poor surgical candidates, however growing evidence suggests that ablation techniques used in combination with radiation, chemotherapy, and surgical management.

### Biliary drain

Percutaneous transhepatic access to the biliary tree offers an alternative route to alleviate malignant obstructive jaundice. Access is traditionally obtained through endoscopic retrograde cholangiopancreatography to drain excess bile and alleviate the obstruction. Currently both options are safe with high success rates and have comparable impact on patient outcomes (79–81). Preferences typically depend more on the hospital system and patient preferences. If the patient has altered surgical anatomy, IR performing biliary endoscopy and drainage may be the only feasible option due to surgically altered anatomy (82).

## Endocrine

Traditionally IR has a limited role in treating endocrine related diseases. Recent experimental studies are investigating the utilization of thyroid artery embolization for the treatment of Graves’ disease and multinodular goiter (83). This therapeutic option is high risk with several reported complications including thyroid storm and cerebral infarctions (84). With proper patient selection and technique the procedure can be safe and effective for the treatment of nodular goiter to reduce thyroid volume and thyroid hormone (85). This technique is still under investigation and is not typically recommended unless patients are poor surgical candidates or fail other therapy.

Venous sampling can be an important procedural step when prior imaging by ultrasound and Tc99 scintigraphy fail to adequately localize the source of hypertrophy or hormone secreting tumor. IR physicians can be instrumental in gaining access to the venous drainage of the parathyroid, petrosal sinus of anterior pituitary, adrenal glands, or pancreatic veins of islet cells. This is often a secondary intervention due to additional financial and procedural exposure that would otherwise be avoided with proceeding surgery. In cases where imaging was indeterminate in hyperthyroidism, venous sampling could identify the calcitonin hormone reliably to improve management (86).

For primary aldosteronism, venous sampling has become an important diagnostic step in identifying unilateral or bilateral adrenal disease (87). By using CT and MR alone, there is poor ability to detect laterality of disease (88). Despite incidental adenomas, there is controversy about the utility after the SPARTACUS trial demonstrated similar outcomes to CT alone at one year. Regardless, the practice of adrenal sampling is the gold standard to confirm the source of hormonal secretion (89). Similarly, utilizing pancreatic venous sampling with calcium stimulation can improve the location of endocrine tumors better than angiography alone (90).

## Lymphatics

Magnetic resonance lymphangiography plays a key role in various clinical scenarios. Most often there is a need to visualize lymphatic flow and structures such as the thoracic duct or cisterna chyli in plastic bronchitis, chylothorax, chyloperitoneum, and intestinal lymphangiectasia (91). In patients with postoperative lymphatic leaks, lymphangiography with sclerosing oils can be sufficient for reducing the leak (92). Larger leaks involving the thoracic duct can undergo embolization in lieu of surgical ligation with high rates of success without exposing the patient to surgical risks.

In the settings of cancer, visualization of the lymphatics can also help assess staging. Incurring the initial costs of obtaining the imaging can more appropriately stage breast cancer and maximize life expectancy and life extended per dollar spent (93). Similarly, lymphatic imaging can be utilized for proper staging in abdominal lymphomas, though PET-CT scans have become standard due to their increased resolution capabilities (94, 95). Thus, this is another tool in the IR kit to provide to oncologists to better manage complex cancer patients.

## Gastrointestinal

### Gastrointestinal stent placement

Gastroduodenal stent placement for gastroduodenal obstruction is a therapeutic option for malignant strictures or benign obstruction with other failed therapeutic trials (96). The role of IR for percutaneous placement is often addressed if transoral endoscopic options fail or are poor surgical candidates. The goal of stent placement is to resolve the obstruction and improve quality of life. Percutaneous access yields a high rate of success when performed via transhepatic access if biliary stents are required. IR is well suited for this procedure that can offer quick resolution of symptoms in a cost-effective manner (97). While surgical gastrojejunostomy remains the gold standard for the longest resolution of symptoms, stenting is a therapeutic option that may be beneficial for oncology patients.

IR can also provide obstructive relief for colonic obstruction (96). Endoscopic stent placement is often sufficient in distal obstruction, but factors can prevent adequate access to proximal colonic obstruction that would necessitate urgent surgical intervention. IR can provide a last resort option for fluoroscopic stent placement using wires through the anus with a high level of success in relieving obstruction (98, 99).

### Percutaneous radiological gastrotomy (PRG)/gastrojejunostomy/jejunostomy

IR physicians are skilled at placing percutaneous radiological gastrotomy and gastrojejunostomy tubes for enteral nutrition (100). Radiologic placement has proven to be as safe and effective as endoscopic placement with a reduced risk profile relative to surgical placement (101). Whether IR places the gastrotomy tube or not usually depends more on the institutional resources or contraindications, as no clear advantages have been established (102). The comparative costs can also be quite variable, though Percutaneous endoscopic gastrotomy (PEG) is either of similar or higher cost as this is more frequently done in an operating room suite (103). In terms of average CMS reimbursement rate, the 2022 facility CPT codes currently list PEG and PRG tube placement to be $203 and $204 respectively.

## Renal

### Nephrostomy/nephro-ureteral tube & stent placement

IR physicians are instrumental in the treatment of urinary obstruction. Interventional oncology is frequently consulted as chemotherapies, radiation, and tumors are common etiologies of obstruction. IR use of ultrasound and fluoroscopy allow for high fidelity placement and replacement of nephrostomy tubes. IR performs a significant volume of nephrostomy tubes, comprising over 90% of nephrostomy tube claims according to Medicare data (104). When percutaneous access is gained, IR can do simultaneous pressure measurements with the Whitaker test to complement diuretic renography. Compared with nephrologists or urologists that may be achieving access blind or with ultrasound, the fluoroscopic feedback allows for safer and more cost-effective placement. Urology is more often the operator for the placement of nephro-ureteral tubes and ureteral stents (104). When stent placement requires retrograde access, urology has more expertise to operate, but IR can use similar methodology when tube and stent placement is delivered anterograde with percutaneous access.

### Nephrostolithotomy

With large obstructive stones, percutaneous nephrostolithotomy remains the most efficacious technique (105). The stone can be disintegrated such that small pieces can be removed from a dilated nephrostomy tract. Nephrostolithotomy staghorn calculi lead to faster recovery times, fewer infections, and with longer stone free periods (106, 107). Pediatric populations also benefit from this procedure for stone resolution (108). This technique while carrying nontrivial risks of bleeding, an experienced IR physician can minimize complications.

### Suprapubic cystostomy

Prolonged bladder outlet obstruction leads to hydronephrosis, kidney failure, and parenchymal fibrosis. In pelvic trauma, posterior urethral injury is a frequent occurrence that prevents foley catheter placement. While attempts are made for early primary realignment, the gold standard to treatment is cystostomy with delayed urethroplasty (109, 110). There is also evidence that percutaneous suprapubic cystostomy by IR may be preferable to repeated catheterization or surgical cystostomy (111). Even in clinical scenarios requiring short-term bladder drainage, suprapubic catheters had less bacteriuria, pain, and recatheterization without reports of elevated risks or complications (112, 113). These benefits to patients are also seen when replacing indwelling catheters, with fewer catheter associated urinary tract infections (114). IR operators are key to keeping complication rates low to make this alternative equally safe to perform.

### Renal denervation

Ablation of the nerve plexus of the renal artery is an interventional technique to address resistant hypertension (115). Randomized control trial SIMPLICITY HTN-2 demonstrate efficacy in reducing systolic blood pressure, however SIMPLICITY HTN-3 which included a sham control trial did not show any added benefit (116). The SYMPATHY trial did not show benefits over medication alone, though medication compliance was inconsistent between trial arms (117, 118). While the procedure has been demonstrated to be safe in all trials, the efficacy of the procedure with current techniques lacks the evidence necessary that it is an effective form of hypertensive management.

## Integumentary

Cosmetic procedures have been a growing and lucrative field of medicine that has been a growing frontier for IR (119). IR focuses on vascular anomalies that cause undesirable dermatologic changes such as vascular malformations, varicose veins, and spider veins. Cutaneous vascular malformations can be embolized successfully in similar fashion to other vascular procedures where surgical removal is unnecessary or infeasible (120). Varicose vein treatment has been transformed by the rise of percutaneous interventions, where surgical ligation and stripping can be substituted with laser ablation or radiofrequency ablation with similar efficacy (121). There is a dearth of available evidence regarding specialty specific performance, but IR is well suited with the skills to manage these patients.

## Reproductive

### Uterine fibroid embolization

Embolization of large uterine fibroids has been utilized in the treatment algorithm for uterine-preserving conservative medical management. The procedure has been gaining in relative popularity compared to myomectomy and endometrial ablation (122). Various studies have shown that fertility can also be conserved in the majority of patients despite potential compromise to endometrial vasculature and radiation exposure (123). Thus, embolization competes for utility among myomectomy and hysterectomy as the definitive treatment of abnormal uterine bleeding. While there is a general increase in the re-treatment for embolization, they generally have great responses to the procedure with minimal complications and a shorter hospital stay relative to either procedure (Table 1).

**Table 1:** Review of comparative studies of IR procedures. Only comparative studies on IR procedures investigating surgical or medical alternatives are included in this review.

Procedure	Reference	Comparison	Method	Statistically significant findings
Whole body (systemic)				
Biopsy	Hassan, et al., 2012 (124)	Surgical biopsy	Retrospective	Needle biopsy had fewer complications than open biopsy for neuroblastoma
Biopsy	Liberman, et al., 1998 (125)	Surgical biopsy	Cohort	US-biopsy obviated need for surgical biopsy, which subsequently reduced cost (older paper)
Biopsy	Luparia, et al., 2011 (126)	Surgical biopsy	Retrospective	Breast vacuum assisted needle core biopsy obviated surgical biopsy, w/ significant cost savings
Biopsy	Fernandez-Garia, et al., 2017 (127)	Surgical biopsy	Retrospective	Needle core biopsy obviated surgical biopsy, w/ significant cost savings and similar diagnostic effectiveness
Biopsy	Thompson, et al., 2018 (128)	Surgical biopsy	Randomized control trial	MRI guided prostate biopsy increased accuracy with minimal relative cost increase
Biopsy	Aggarwal, et al., 2019 (129)	Surgical biopsy	Retrospective	IR obtained more, higher quality biopsy samples
Biopsy	Smith, et al., 1995 (130)	Surgical biopsy	Retrospective	Outpatient liver biopsy was safe and cost effective
Biopsy	Bruening, et al., 2010 (131)	Surgical biopsy	Systematic review	Core biopsy safer than open surgical, with equal quality
Drains	Hemming, et al., 1991 (132)	Surgical drainage	Retrospective	Drain vs. surgical outcome equivalent for diverticular abscess
Aspiration	Cinat, et al., 2002 (133)	Surgical drainage	Prospective cohort	Intra-abdominal infections were effective with a single treatment in 70% of patients and increased to 82% with a second attempt
Pre-operative tumor marking	Shaikh, et al., 2010 (134)	No marking	Retrospective	Tumor marking significantly improved visualization score and clinical targeting for lumpectomy
Tumor markings	Suzuki, et al., 1999 (135)	No marking	Retrospective	Indications of failure for nodule, indicating need for marking
Trauma & hemorrhage hemostasis	Asensio, et al., 2003 (20)	Surgical vs. IR initial intervention	Retrospective	Angioembolization decreases mortality in grade IV, V hepatic injuries
Vascular				
Implantable venous access device insertion	Hancock-Howard, et al., 2010 (136)	Surgical placement	Retrospective cohort	IR placement in pediatric patients was cheaper than operative placement
Tunneled central venous catheter	Stevenson, et al., 2002 (137)	Nontunneled	Retrospective	Infection rate 3 × higher
Tunneled central venous catheter	Weijmer, et al., 2004 (138)	Nontunneled	Retrospective	Complication rates higher at 2 weeks
IVC filter placement & removal	Makary, et al., 2018 (38)	IR vs. OR placement/retrieval	Retrospective cohort	IR used half the fluoroscopy time vs. OR (*P* = 0.02) for filter removal, direct costs of OR 20% > than IR (*P* = 0.01) filter placement
Hepatobiliary				
Tunneled peritoneal catheter	Ozener, et al., 2001 (65)	Retrospective	Percutaneous vs. surgical	Fewer removals and increased 1- and 2-year overall survival
BRTO	Ozman, et al., 2022 (31)	Meta-analysis	BRTO vs. beta blocker	BRTO lowered risk of rebleeding in patients with cirrhosis and a previous gastric varix bleed
TIPS	Papatheodoridis, et al., 2003	Meta-analysis	Endoscopic treatment vs. TIPS	Significant reduction in rebleeding rates, but a significantly higher risk of inducing encephalopathy
TIPS	Korsic, et al., 2021 (70)	Retrospective	Endoscopic treatment vs. TIPS	TIPS is more effective at preventing rebleeding, but does not increase overall survival
TIPS	Halabi, et al., 2016 (69)	Meta-analysis	Endoscopic treatment vs. TIPS	Prophylactic TIPS prior to major variceal bleed increases 1 year survival
TIPS	Nicoara-Farcau, et al., 2021 (71)	Meta-analysis	Endoscopic treatment vs. TIPS	Preemptive TIPS with Child-Pugh score B and C and bleeding increases 1 year survival
Biliary drainage	Artifon, et al., 2012 (139)	Randomized control trial	Percutaneous vs. endoscopic drain	Percutaneous drainage and EUS-CD were comparable in in success and complication rates
Biliary drainage	Liu, et al., 2018 (79)	Meta-analysis	Percutaneous vs. endoscopic drain	Both had similar success rate metrics with fewer short term postoperative complications
Biliary drainage	Dorcaratto, et al., 2018 (81)	Meta-analysis	Percutaneous vs. endoscopic drain	Percutaneous drains had fewer complications in patients awaiting pancreaticoduodenectomy
Endocrine				
Parathyroid venous sampling	Haciyanli, et al., 2021 (86)	Parathyroid imaging	Retrospective	Patients with ambivalent imaging were able to identify hormonal source in most patients
Inferior petrosal sinus sampling	Radvany, et al., 2016 (140)	IJ sampling	Retrospective	IJ sampling was inferior/insufficient compared to petrosal sinus sampling
Adrenal venous sampling	Ladurner, et al., 2017 (88)	CT and MRI alone	Retrospective	CT and MR have poor unilateral detection without venous sampling
Islet cell tumor venous sampling	Wiesli, et al., 2004 (141)	CT/MR and surgery	Retrospective	ASVS is the most sensitive technique for tumor identification
Islet cell tumor venous sampling	Roche, et al., 1982 (90)	Arteriography	Retrospective	Pancreatic venous sampling was highly effective at tumor localization
Musculoskeletal				
Vertebroplasty	Klazen, et al., 2010 (57)	Medical management	Randomized control trial	Pain was reduced after one month and one year
Geniculate artery embolization	Lee, et al., 2019 (142)	Medical management	Prospective cohort	Pain was significantly reduced from baseline up to 6 months after procedure
Geniculate artery embolization	Bagla, et al., 2020 (143)	Medical management	Prospective cohort	Pain was significantly reduced from baseline up to 6 months after procedure
Geniculate artery embolization	Little, et al., 2021 (144)	Medical management	Prospective cohort	Pain and synovitis were reduced from baseline up to 1 year after procedure
Geniculate artery embolization	Okuno, et al., 2017	Medical management	Prospective cohort	Pain was reduced from baseline up to 24 months with a reduction of synovitis
Geniculate artery embolization	Okuno, et al., 2014	Medical management	Prospective cohort	Pain was significantly reduced from baseline 1 and 4 months after procedure
Percutaneous kyphoplasty	Kasperk, et al., 2010 (145)	Medical management	Prospective cohort	Patients had one-year improvements in pain and mobility
Percutaneous kyphoplasty	Garfin, et al., 2006 (146)	Medical management	Prospective cohort	Patients had improved pain, functional and mental outcomes up to 2 years
Percutaneous kyphoplasty	Wardlaw, et al., 2009 (147)	Medical management	Randomized control trial	Patients had improvements from baseline up to 2 months after procedure
Percutaneous kyphoplasty	Schmelzer-Schmied, et al., 2009 (59)	Medical management	Retrospective cohort	Patients had significant improvements in pain and mobility
Sinopulmonary				
Bronchial stenting	Li, et al., 2014 (148)	IR airway placement with biopsy	Retrospective cohort	IR was able to place airway stents safely and successfully with biopsy
Thermal ablation	Kwan, et al., 2014 (149)	Resection vs. thermal ablation	Retrospective	Lobe resection and ablation had equal outcome
Lymphatic				
Thoracic duct embolization	Itkin, et al., 2010 (150)	Surgical ligation	Prospective cohort	Procedure was safe and feasible with a high success rate
Therapeutic lymphangiography	Alejandre-Lafont, et al, 2011 (151)	Conservative management	Prospective cohort	70% saw a statistical decrease in effusion volumes less than 500 ml/day
Intranodal lymphangiography	Yannes, et al., 2017 (152)	Thoracic duct embolization	Prospective cohort	Injection of sclerosing agents intranodally has similar success to embolization
Gastrointestinal				
Gastroduodenal stenting	Maetani, et al., 2004 (153)	Surgical bypass	Retrospective	Quicker improvement of obstructive symptoms and performance
Gastroduodenal stenting	Mittal, et al., 2004 (154)	Open and laparoscopic gastrojejunostomy	Retrospective	Reduced time to oral intake, hospital stay, and complications
Gastroduodenal stenting	Jeurnink, et al., 2007 (155)	Gastrojejunostomy	Retrospective	Reduced time to oral intake, hospital stay. Higher rate of reintervention
Gastroduodenal stenting	Jeurnink, et al., 2010 (97)	Gastrojejunostomy	Randomized trial	Faster food intake, but a faster recurrence of obstructive symptoms and shorter life expectancy
Gastroduodenal stenting	Park, et al., 2016 (156)	Gastrojejunostomy	Retrospective	Faster dysphagia improvement and shorter hospital stay, but with a higher recurrence rate
Colonic stenting	Yoon, et al., 2017 (98)	Failed endoscopic placement	Retrospective	Right sided colonic obstruction relieved by fluoroscopic guidance avoiding emergency surgery
Colonic stenting	Kim, et al., 2020 (99)	Failed endoscopic placement	Retrospective	Obstructive resolution was achieved with high rate of success (93%)
PRG	Wollman, 1995 (101)	PEG	Meta-analysis	PRG and PEG are equivalent in safety and efficacy with fewer risks than surgical placement
PRG	Galaski, et al., 2009 (157)	PEG	Retrospective	PEG had longer length of stay
Renal				
Renal denervation	Geisler, et al., 2012 (158)	Antihypertensives	Markov model (Simplicity HTN-2 trial)	Renal denervation reduced 10-year risk of stroke, MI, HF, ESRD, and all coronary heart disease
Renal denervation	Tilden, et al., 2014 (159)	Antihypertensives	Meta-analysis	Increased quality of life years, but at greater cost per year
Suprapubic cystostomy	McPhail, et al., 2006 (112)	Transurethral catheterization	Meta-analysis	Suprapubic catheters had less incidence of bacteriuria and less discomfort, with no change in repeat catheterizations
Suprapubic cystostomy	Niël-Weise, et al., 2005 (113)	Indwelling urinary catheter	Systemic review	Suprapubic catheters had less incidence of bacteriuria, discomfort, and fewer repeat catheterizations
Suprapubic cystostomy	Gibson, et al., 2019 (114)	Indwelling urinary catheter	Prospective cohort	Fewer urinary tract infections, hospitalizations, and antibiotic use. Cystostomy infections had more drug resistant organisms.
Percutaneous nephrostolithotomy	Meretyk, et al., 1997 (106)	Shock wave lithotripsy	Prospective cohort	Nephrostolithotomy with lithotripsy was more successful with fewer infections, shorter treatment length, and fewer complications
Percutaneous nephrostolithotomy	Lingeman, et al., 1994 (107)	Shock wave lithotripsy	Meta-analysis	Nephrostolithotomy with lithotripsy was more successful
Reproductive				
Uterine fibroid embolization	Mara, et al., 2006 (160)	Myomectomy	Randomized control trial	UFE had shorter procedure length, hospital stay, disability, CRP, and higher hemoglobin 48 h after surgery, at the cost of higher re-intervention rate and less consistent symptom relief
Uterine fibroid embolization	Pinto, et al., 2003 (161)	Hysterectomy	Randomized control trial	Hysterectomy and UFE had similar safety profile, but with a 4-day shorter hospital stay
Uterine fibroid embolization	Razavi, et al., 2003	Myomectomy	Retrospective	UFE was better for menorrhagia but worse for mass effect. UFE had fewer complications, shorter hospitalization, and fewer days of narcotics.
Uterine fibroid embolization	Siskin, et al., 2006 (162)	Myomectomy	Prospective	UFE had similar outcome, but with increased QOL and fewer adverse events
Uterine fibroid embolization	Jun, et al., 2012 (163)	Myomectomy and hysterectomy	Randomized control trial	UFE had higher minor complications, readmissions, but a shorter hospital stay
Uterine fibroid embolization	Hehenkamp, et al., 2005 (164)	Hysterectomy	Randomized control trial	UFE had shorter hospital stay, a shorter recovery time, and less major complications.
Varicocele sclerotherapy	Cayan, et al., 2013 (165)	Open and laparoscopic varicocelectomy	Meta-analysis	Embolization was comparable in efficacy and fertility recurrence to surgical approaches
Varicocele sclerotherapy	Abdulmaaboud, et al., 1998 (166)	Open and laparoscopic varicocelectomy	Retrospective	Sclerotherapy was shown to have lower complication rates than open surgery with comparable increases in sperm motility rates
Prostatic embolization	Ray, et al., 2018 (167)	TURP	Prospective cohort	Patients had shorter hospital stay and fewer readmissions, but was not as robust as TURP
Prostatic embolization	Zumstein, et al., 2019 (168)	Surgical therapies	Meta-analysis	PAE had fewer side effects, complications, or impact on sexual function. Symptomatic improvement not as robust

All statistically significant findings in each study are addressed. Case studies are excluded.

### Fallopian tube recanalization

Fallopian tube obstructions are caused by tubal spasms, pelvic inflammatory disease, endometriosis, polyps, congenital malformations, salpingitis isthmica nodosa, or intratubal debris is a common primary or secondary etiology in in infertility. Proximal obstructions are easily accessible for recanalization. Hysterosalpingography can successfully identify the origin of the obstruction and catheterization past the obstruction can be sufficient to provide clear passage to the infundibulum. Successful pregnancy and delivery rates have been reported as a result (169, 170).

### Varicocele sclerotherapy

The therapeutic options for varicoceles that are painful or causing a reduction in fertility include open and laparoscopic varicocelectomy or anterograde/retrograde sclerotherapy (171). When comparing embolization to surgical options, there is a comparable alleviation of symptoms and increase in fertility rates (165). Sclerotherapy has an advantage in that this can be done quickly and minimally invasively, and therefore have fewer associated costs (172). Surgical options may be better when dealing with severe cases to prevent higher rates of reintervention.

### Penile venoablation and angioplasty

Vascular causes of erectile dysfunction can be addressed by IR physicians. In cases of arterial claudication, the delivery of drug-eluting stents in the internal pudendal arteries or common penile arteries can restore erections. In venous pathology, embolization of the dorsal vein can also regain functional erections using ethanol, coils, or balloons (173–177). For patients with severe dysfunction due to venous leakage resistant to medication, endovascular therapeutics are often the best option.

### Prostate embolization

There are several potential treatments for benign prostatic hyperplasia (BPH) including medical management, surgical transurethral resection (TURP), laser vaporization, and open prostatectomy. There is a major complication risk of causing erectile and ejaculatory dysfunction as a result (178). Prostatic artery embolization does not carry a similar risk, while also being comparably effective for BPH treatment (179). Compared to surgical options, prostatic embolization had faster recovery times and fewer complications, but was not as robust in alleviating symptoms (167). IR can offer a therapeutic option with fewer risks and symptom relief in milder cases that have failed medical management.

## Medical and financial value of minimally invasive procedures

Training in minimally invasive procedures equips the IR physician with a wide toolset in techniques and equipment that enables intervention throughout the whole body. IR consults with specialists ranging from gastroenterologists, nephrologists, gynecologists, urologists, pulmonologists, and neurologists. IR also has advanced relationships with oncologists, serving as a consultant for disease diagnosis, central venous access, monitoring, and therapeutic procedures. There has been a concerted effort in the field to demonstrate the value added to medical care through these procedures in terms of patient outcomes.

### Therapeutic value

The procedures performed in IR are valuable to many medical subspecialties. There are many procedures exclusive to IR that offer alternative minimally invasive options to surgical or medical intervention. There is a large library of evidence that these IR procedures provide comparative advantages to these surgical and medical management (Table 1). When there are cross-specialty operators, IR's specialty training can yield better results than alternatives including biopsies, pediatric implantable venous access devices, and IVC filter placement (38, 129, 136). IR's performance in biopsy has made them the predominant operator for all biopsies at most hospital systems over the past 20 years.

Comparative studies and meta-analyses of IR procedures show common therapeutic benefits. Patients have shorter recovery times, commonly due to pain being successfully managed with local anesthetics and moderate sedation alone facilitating timely discharges. Procedures have fewer complications and infections, because even laparoscopic surgery has more sites of bacterial access and risk of damaging internal structures. Procedures can be more robust than conservative medical management, and while procedures like geniculate artery embolization, renal denervation, kyphoplasty, and nephrostolithotomy do introduce bleeding and infection risk, they offer more robust effects without exposing the patient to the risks of full surgical management. In interventional oncology, palliative procedures are faster and safer than surgery.

IR procedures do have their limitations, in that many times their benefits are often temporary. Surgery for uterine fibroids or prostatic hyperplasia offers definitive resolution, where embolization may require repeat trials. In palliative care, placing stents in bronchial tree or within the GI tract may provide immediate relief, but the tumor's growth will quickly obstruct the stent and require surgery. Patients must then choose between a permanent and expedient solution. The value of offering choice to the patient and specialist to treat their illness cannot be understated. The heterogeneity of presentation and physician preferences allow for greater future optimization of patient care and comfort than ever before.

### Financial value

The ability of IR to reduce costs is beneficial to all the healthcare stakeholders. There have been studies comparing the financial differences between IR procedures and surgical procedures that generally show greater financial value to patients, hospitals, and payers (Table 2). Similarly, using IR physicians for procedures where they are most competent also saves money relative to other operators (38, 199). However, these benefits may be limited to the organizational structure of the hospital system and reimbursement model (202, 203).

**Table 2:** Review of comparative economic studies of IR procedures. Only comparative studies on IR procedures investigating surgical or medical alternatives are included in this review.

Procedure	Reference	Comparison	Method	Statistically significant findings
Whole body (systemic)				
Biopsy	Brownleee, et al., 2020 (180)	Inpatient vs. outpatient	Retrospective	Safe and cost effective for outpatient procedure
Biopsy	Na, et al., 2020 (181)	Surgical biopsy	Retrospective	Percutaneous biopsies were cheaper w/ reduced hospital stays vs. surgical
Biopsy	Pistolese, et al., 2012 (182)	Surgical biopsy	Retrospective	Breast surgical biopsy cheaper vs. vacuum assisted biopsy w/ equal diagnostic value
Biopsy	Lachar, et al, 2007 (183)	Surgical biopsy	Retrospective	Needle core is cheaper than open biopsy for lymphomas
Biopsy	Silverman, et al., 1998 (184)	Surgical biopsy	Retrospective	Abdominal biopsy cheaper than surgical
Biopsy	Tsai, et al., 2020 (185)	Surgical biopsy	Retrospective	Breast surgical biopsy cheaper vs. vacuum assisted biopsy w/ equal diagnostic value
Biopsy	Sutton, et al., 2013 (186)	Surgical biopsy	Model based systematic review	Lymph node biopsy cost effective and equal outcome to inguinofemoral lymphadenectomy
Biopsy	Gruber, et al., 2008 (187)	Surgical biopsy	Meta-analysis	Needle core is cheaper than open biopsy
Drains	Botana-Rial, et al., 2021 (188)	Paracentesis	Systematic review	Indwelling catheter for malignant effusion increased quality of life in cost effective manor
Vascular				
Chest port insertion	LaRoy, et al., 2015 (189)	IR vs. OR suite placement	Retrospective	IR suites were cheaper with comparable OR suite placement outcomes
Implantable venous access device insertion	Hancock-Howard, et al., 2010 (136)	Surgical placement	Retrospective cohort	IR placement in pediatric patients was cheaper than operative placement
TIVAD	Martin, et al., 2022 (190)	OR vs. IR	Retrospective	TIVAD placement was 16% more expensive on average in OR vs. IR (*P* < 0.01)
IVC filter placement & removal	Makary, et al., 2018 (38)	OR vs. IR	Retrospective cohort	IR used half the fluoroscopy time vs. OR (*P* = 0.02) for filter removal, direct costs of OR 20% > than IR (*P* = 0.01) filter placement
Splenic artery embolization	Kanters, et al., 2021 (191)	Embolization vs. splenectomy	Retrospective cohort	Embolization intervention was more cost effective and increased QALY in splenectomy
Hepatobiliary				
Transjugular intrahepatic portosystemic shunt (TIPS)	Russo, et al., 2000 (72)	Endoscopic sclerotherapy	Retrospective	TIPS was more cost effective per bleed with lower recurrent bleed rates
Radiofrequency ablation	Cucchetti, et al., 2013 (192)	Surgical resection vs. RFA	Markov model	For a single lesion <2 cm or multiple <3 cm, RFA was the most cost effective therapeutic.
Radiofrequency ablation	Spolverato, et al., 2015 (193)	Surgical resection vs. RFA vs. transplant	Cost analysis	Milan criteria and Child-Pugh A cirrhosis patients with RFA or resection had more cost-effective outcomes than transplantation
Tunneled peritoneal cavity	Bohn, et al., 2015 (68)	Paracentesis	Sost-minimization	Peritoneal catheter saves costs for malignant ascites after an average of 83 days
Tunneled peritoneal cavity	Sennafalt, et al., 2002 (194)	Hemodialysis	Decision tree model	The cost per quality adjusted life year was less for peritoneal dialysis
Endocrine				
Parathyroid venous sampling	Sato, et al., 2015 (195)	Markov modelling	Standard workup	The costs of adding venous sampling to complete workup did not improve outcomes and increased cost
Adrenal venous sampling	Lubitz, et al., 2015 (196)	Decision tree model	CT/MR in resistant hypertension	CT/MR plus venous sampling was the most cost-effective screening approach
Musculoskeletal				
Percutaneous kyphoplasty	Itagaki, et al., 2012 (58)	Surgery	Retrospective	Reduced cost and length of stay versus surgery
Sinopulmonary				
Thermal ablations	Kwan, et al., 2014 (149)	Lobar resection NSCLC	Retrospective cohort	Costs were significantly less for thermal ablation due to outpatient setting
Lymphatic				
Lymphangiography	Pandharipande, et al., 2008 (93)	Sentinel biopsy staging in breast cancer	Markov modelling	The addition of lymphangiography best increased quality adjusted life expectancy per medical dollar spent
Gastrointestinal				
Gastroduodenal stenting	Jeurmink, et al., 2010 (97)	Gastrojejunostomy	Randomized control trial	Stenting was cheaper than gastrojejunostomy
PRG	Myssiorek, et al., 1998 (197)	PEG	Retrospective	PEG was over 500% more expensive than PRG
PRG	Barkmeier, et al., 1998 (198)	PEG and SEG	Retrospective	PEG < PRG < SEG. PEG and PRG equivalent for GJ and less than SEGJ
Renal				
Hemodialysis access maintenance	Trivedi, et al., 2020 (199)	Nephrologist and surgeon placement	Longitudinal cohort study	Nephrologists and Surgeons were 59% (*P* < 0.001) and 57% (*P* < 001) more expensive than IR
Renal denervation	Tilden, et al., 2014 (159)	Antihypertensives	Meta-analysis	Increased quality of life years, but at greater cost per year
Renal denervation	Geisler, et al., 2012 (158)	Antihypertensives	Markov model (Simplicity HTN-2 trial)	Cost savings accumulate for each quality-of-life year gains from reduced CV events
Reproductive				
Varicocele sclerotherapy	Abdulmaaboud, et al., 1998 (166)	Open and laparoscopic varicocelectomy	Retrospective	Sclerotherapy was significantly cheaper than open and laparoscopic surgery
Varicocele sclerotherapy	Johnsen, et al., 1996 (172)	Surgical varicocelectomy	Retrospective	Anterograde sclerotherapy is cheaper than all surgical options
Uterine fibroid embolization	Cain-Nielsen, et al., 2014 (200)	MR-focused ultrasound and myomectomy	Markov modelling	All three treatments offered similar QALY benefits, but UFE was most cost effective
Uterine fibroid embolization	Volkers, et al., 2008 (201)	Hysterectomy	Retrospective (EMMY Trial)	UFE had significantly lower mean total costs and saved patient costs with reduced work absences

All statistically significant findings in each study are addressed. Case studies are excluded.

The common cost saving measures of IR procedures are the facilities. IR suites are typically less expensive than OR suites. Similarly, local anesthetic and moderate sedation is cheaper than having an anesthesiologist present. Because there is less anesthesia and manageable pain, these patients can confidently be outpatient, saving on overall patient costs. IR procedures also achieve greater patient value over medical management because they can be more effective at treating disease over long-term analysis of cost and quality adjusted life years. Collectively, the data demonstrate the utility of the IR physician to be a value driven member of the medical team.

### Research and innovation

IR is a young field with a prominent history of procedural and technical innovation. IR's success has been centered on the ability to develop new less invasive strategies to benefit patients. Innovation is one of the cornerstones of IR, with physician motivation, their fascination with novelty, venturesomeness, and mentorship have made this specialty one of leading drivers of change in medicine under the principle that, “less is more” (204). Because of IR's practice touching most other specialties in medicine, they are well positioned to both understand and identify limitations in the management of disease processes and imagine solutions without the burdens of conventional dogma (205).

The current areas of innovation in the field focus on new applications for existing procedures. New “embotherapies” are novel applications of localized ischemia or necrosis in disease treatment (206). Similarly, immuno-oncology will utilize IR for localization of new targeted therapies for chemotherapy delivery and gene therapies. IR is also exploring new ways to visualize their procedures. The expansion of tools available for virtual reality and augmented reality allow further evolution on how anatomy is displayed during the procedure (207). With new visualization comes with new tools for recognition and with the growth of artificial intelligence tools in radiology, there is a growing need to focus efforts on applying these tools to high yield areas (208).

While the initial development of IR procedures was overshadowed by other specialties adopting these techniques, the future is optimistic for the expansion in their value to medicine. The preliminary techniques were easily adaptable relative to open surgery, but the evolution of advanced tools to challenge the limitations of image guided micro procedures will require advanced nontransferable expertise. Novel IR procedures introduced are often initially expensive and difficult to garner support for innovation at an institution. However, advocating for the long-term impact of new procedures that decrease morbidity, mortality, and overall cost will reinforce the goals of modern healthcare systems to reduce healthcare expenses.

## Financial contributions

### Direct contributions

IR makes direct contributions to patient care. Diagnostic and therapeutic interventions are directly reimbursed by healthcare payers. These procedures generate billable relative value units (RVU) that go toward directly reimbursing the facilities, labor, and equipment costs. The forces guiding these procedures are not only guided by internal factors of the skills of the practitioners in the practice, but also the external needs of the healthcare system and the contractual relationships that are formed (209). These can be quite variable over time, both in volume and in scope of practice. Thus, gaining the appropriate credentialing, equipment, and expertise to serve these obligations best serve patients and generate the most revenue for your responsible parties.

An expanding field for generating revenue has been in the direct contributions in the role of evaluation and management. The utilization of skilled advanced practitioners, such as physician assistants, nurse practitioners, and radiologist assistants, have allowed a longitudinal model to be financially viable with alternative value-based healthcare reimbursement models (210, 211). With their unique billing codes, IR practices can better utilize their physicians for highly skilled tasks, without passing on direct longitudinal clinical activities.

### Indirect contributions

The indirect contributions of IR are the collaborative nature of the field. As the microinvasive proceduralist, they can collaborate with most other specialties in the hospital setting to bring new and specialized procedures for their patients. Because of their efficiency and general low risk, low cost, and faster recovery profiles, patients generally have better experiences that can be passed through their facilities. While most procedures could be technically handled by other specialties, expert operation also frees up the other specialists to focus more on disease management and planning. IR also plays a role in this consultation for diagnostic interpretation, to participate in monitoring and assessment. Furthermore, with IR broadening its public accessibility, IR can also work with other specialties to drive patient flow toward their hospital system. Between the expert consultation, specialist collaboration, procedural efficiency, optimizing patient experience, and directive of patient flow, IR makes the whole healthcare system run more effectively by being a flexible participant in patient care.

### Revenue analysis of IR reimbursement models

The reimbursement paradigm can have a significant impact on the generated revenue for IR physicians, their department, or their practice. While the standard model of reimbursement has historically been a fee-for-service (FFS) model, the implementation of the Patient Protection and Affordable Care Act (ACA) in 2009 has been a driving force for the adoption of alternative reimbursement models. The ACA developed the National Quality Strategy through the creation of the Agency for Healthcare Research and Quality (AHRQ). This agency dictates the payment policy for the Centers for Medicare and Medicaid services (CMS). These services comprise approximately 36% of all national healthcare spending in the United States (212). Over the last decade, the ACA has been progressively shifting toward “alternative payment models” to de-incentivize high throughput service with complications. These include the following: value-based care (pay for performance), accountable care, bundled payment, patient-centered medical home, and capitation. Each of these models operate in parallel in the modern healthcare system and have a distinct impact on how the IR physician can practice.

### Fee-for service model

FFS is still the most common reimbursement model that exists today. Most healthcare organizations today still use this model to generate over 50% of their revenue, and private practices often generating over 75% of revenue from FFS (213). The reimbursement model is based off the current procedural terminology (CPT) codes that assign relative value units (RVUs) to compensate the physician work, practice expense, and professional liability insurance (214). RVUs are adjusted by a geographic practice cost index, and multiplied by the annually updated conversion factor ($34.61 est. 2022) to determine the reimbursement for each CPT. In IR, the bulk of the FSS billing codes are procedural, and heavily rely on these RVUs for compensation. There has been a consistent downtrend in reimbursement rates due to reductions in the conversion factor, as well as reducing the total RVUs for surgical procedures across many surgical subspecialties. IR is no exception, with −2.8% inflation adjusted annual decreases in compensation averaged across common IR procedures (215). This change has had a direct impact on the revenue IR physicians generate when focusing heavily on surgical interventions.

In IR, the RVU rates are frequently not representative of their institutions' realized costs. There is a current shift away from the RVU model for calculation to a Time-Driven Activity-Based Costing model adopted from other industries to approximate costs bottom up (216). When observing ports, biopsies, or clinic visits, a process map is generated through various observational and retrospective analysis to estimate costs for personnel, equipment, and consumables for each event. While a newer development, these studies are currently taking place, such as evaluation of hepatocellular carcinoma treatments for interventional oncology (217). These models highlight how some of the actual costs could be off by over 50% to where the RVU value is currently set (218).

IR physicians have also been impacted by the current FSS schedule for their imaging interpretations, as currently only the most expensive image is reimbursed in full, while every following scan by the same provider on the same day is provided 75% of the standard rate (219). This can be a common occurrence when evaluating for intervention. Similarly, the ICD-10 codes are expansive to allow for diagnostic specificity, but this can also lead to a significant increase in reimbursement delays or denials if coded improperly. Because these codes are frequently evolving and growing, inaccurate coding is one of the most common causes of delayed revenue. There are clear benefits to the IR physician and their group to understand the expected expenses and revenue for different procedures to project gross margin and optimize the efficiency of the team.

### Value-based payment

The value-based reimbursement model was designed to target healthcare waste and reduce the per-capita cost of equivalent healthcare outcomes. In this model, the creation of accountable care organizations are dedicated healthcare professionals that work with providers to investigate current practice to minimize costs while optimizing care. Any shared savings to the Medicare program accrued by participating organizations would then share a proportion of the savings. This has been an opportunity for IR physicians to demonstrate that their procedural expertise will result in significant cost savings that will benefit the healthcare system. There has been an active effort over the past 15 years among radiological societies such as SIR, to ensure that IR physicians have a voice in the restructuring of healthcare payments (220, 221).

There has been an increasing push for more research to demonstrate the financial and clinical value of IR procedures (222). The comparative effectiveness research initiatives have been undertaken to train, publish, and disseminate findings of how IR procedures impact patients and the healthcare system. The American Recovery and Reinvestment Act of 2009 offers extramural sources of funding for physicians to conduct effectiveness research to better inform the implementation of a value-based model. There have since been consensus panels to help guide a standardization of the process for future investigation to ensure findings are reproducible and comparable (223). Specifically, IR costs in healthcare delivery has also been previously addressed in consensus panels to both review past work and further conceptualize how effectiveness research will benefit the field (224). SIR, Radiological Society of North America, and the American Society of Neuroradiology have cosponsored international training efforts to provide IR physicians to have the skills to perform comparative research.

With a shift from an FFS model to a value model, there is increased importance on finding more ways to meaningfully interact with patients outside of an operating room to provide value to the healthcare system. This has been the increasing push for clinical IR practice, where more face to face clinical interactions with the patient provide increased value (8, 222). With the help of advanced practitioners, there is increased revenue to be acquired in a value-based healthcare system. This function in addition to interdisciplinary collaboration has a greater opportunity for monetary reward, especially with prior practices potentially undervalued or simply not compensated in an FFS model (225).

### Bundled payment

The bundled payment mode driven by the Bundled Payments for Care Improvement (BPCI) Initiative is designed to provide a prearranged payment for an episode of care. All services provided to the beneficiaries would be linked to this payment. Depending on the model, this includes prospective or retrospective payment to cover inpatient stay and related services up to 90 days post-hospital discharge. If IR procedures are reimbursed with this model, the practice's gross margin will be dependent on that patients' co-morbidities and procedural risk. Studies of readmission rates for common IR procedures have shown that there is a high 30- and 90-day readmission rate (15%–50%) (226). A set payment will encourage high-quality care to minimize the exposure to unnecessary complications. This may conversely provide perverse incentives to avoid patients with co-morbidities and high-risk procedures because of the large financial downside risk and result in patient neglect.

IR physicians should strive to optimize their utilization of outpatient procedures. For example, angiography can be done safely as an outpatient and reduce the average number of patients filling hospital beds overnight (227). New research into safety should be evaluated periodically to find new ways to expand the value delivered to patients. Similarly, IR physicians have a responsibility to challenge the status quo regarding the use of OR suites when appropriate. For example, placing a femoral venous catheter could be done safely and effectively at a patient's bedside. Cost savings include the facility, time, and improved patient experience through convenience (228). Similarly, applying procedural techniques at bedside for IVC filters, PEG tubes, and dilatational tracheostomy can be done faster, cheaper, and with immeasurable changes in risk for the patient (229–231). An explicit goal of IR includes the discovery of new methodology to improve efficiency in patient care.

### Capitation

The capitation model is when a fixed payment is provided for all necessary patient care costs over a set period. While initially implemented in health maintenance organizations (HMOs) managed by insurance companies, the ACA payment system adopted the system that includes quarterly adjustments for clinical outcomes and patient satisfaction (232, 233). IR has been diversifying the setting in which procedures can be safely performed, including outpatient clinics. If IR physicians offer a therapeutic option that is cheaper and safer than the alternative surgical options, physicians may be in a position where they will be capable to thrive in a capitation model (6).

## Environment for successful practice

For those looking to build or establish a successful department of interventional radiology or private practice, there are factors that should be addressed to fulfill best practice expectations. There have been published standards of management and care that have been proposed as helpful guidance for practitioners in patient management and associated tools required (234). These published sources represent educational tools and not legal standards by which healthcare professionals can be adjudicated. Thus, providing updated and evolving reviews of important parameters is important to continued success of the field.

### Team members

The IR physician is responsible for patient clinical management and procedural performance. Supporting team members include advanced practice providers (APP), nurses, registered radiologist assistant, radiologic technologist, a certified medical assistant, and an administrator. APPs consisting of nurse practitioners and physician assistants have been growing in their scope of practice to facilitate care in both patient care and procedures, and under CMS can bill patients under unique ID numbers. This has been shown to boost productivity for the physician for other tasks (210, 211). With sufficient patient volume, APPs can make a substantial impact on patient care and generate revenue.

Interventional radiology technologists are essential for procedures, with certifications to act as a scrub technician and radiologic technician. They serve the role of a surgical assistant by procurement and organization of surgical tools and wires, while also being proficient in the operation of the C-arm Cone-beam CT scanner, powered injectors, and associated software. The addition of a float technician to retrieve additional tools is required as many procedures are dynamic and are best suited to communicate and retrieve the proper equipment. Similarly Registered Radiologist Assistants can provide similar value from the imaging services. Under supervision, they can perform tasks related to patient management, assessment, and preliminary imaging observations. They can help protocol or coordinate with medical imaging technologists to streamline the process of acquiring proper imaging studies of diagnostic quality.

Nurses serve essential functions as a liaison of patient care and communication. For operations, they can gather history, screening, and vitals. For operations requiring sedation, they provide medications and monitor the patient's status. Lastly, they can follow-up with the patient regarding education, wound management, and updating family members. The managerial role of coordinators includes triage referrals, assist with research protocols, and general scheduling or consultations. Certified medical assistants are an adjuvant for nursing functions. These individuals require less advanced education and certifications to provide basic functions that may become challenging due to the volume of activities performed if there is high patient volume. IR teams should staff reflexively to procedural demand and have feedback from nursing staff accordingly to ensure the safety and completeness of patient care.

Lastly, administrative functions are extremely important and responsible for scheduling, precertification, procedural coding, claim submission, structured reporting, and quality improvement. While these functions are fluid, physicians need to provide training in these operations to ensure limited down-time or interruptions in reimbursement. The administrative burden will vary based on inpatient or outpatient clinical practice, and the IR physician can prospectively plan to the expected workload.

### Facility requirements for clinical and operational practice

To provide interventional procedures, an operating room with the addition of imaging equipment creates a hybrid operating space. Several guidelines have been published and construction is usually dictated by local regulatory standards, and hybrid models for minimally invasive procedures have proven to be safe and efficient (235). Specific equipment that are important for most vascular procedures include: large image monitor, Cone-beam CT scanner with biplane imaging and 3-D angiography, C-arm compatible table, pressure injectors, and ultrasound. For biopsy procedures availability of a wide bore CT-scanner with CT fluoroscopy can prove to be beneficial based on physician preference and patient size. With this equipment, software functions involve image capture, image modification, and digital compatibility with the picture archiving and communication system (PACS). Radiation safety equipment is also required, including radiation dosimeter, lead vests with thyroid cover and lead glasses, and radiation shields proven to reduce scatter exposure (236–238). With procedures requiring moderate sedation or general anesthesia, there should also be a post-anesthesia recovery space equipped appropriately to manage complications associated with sedation (239).

In the clinical or outpatient setting, the requirements needed for practice are less specialized than other medical practices. Most of the consultation and follow-up functions can be served by standard clinical offices. Additional tools important for function may also include the close access of ultrasound machines with doppler, vein light, high resolution monitors with multiple displays, dictation/transcript capabilities, and PACS integration.

## Physician recruitment and retainment

If you are building a practice or department, you will need to recruit new IR physicians in a competitive market. Thus, these elements are important for your team to communicate to the IR community such that interested qualified candidates would be interested in joining your team (Figure 3). Beyond entering your team, you want them to be satisfied and be a part of the future growth of that team.

### Expectations

IR daily activities can be highly variable and dependent on their training and the priorities of the hosting institution. All parties would benefit from clarity in teaching obligations, group governance, mentoring, incentives, benefits, and scope of practice. Having a detailed conversation about the details of the job obligations will guide performance and satisfaction from the physician. When discussing the clinical duties, there should also be details for logistics including patient population, case logs, call schedule, case mix, and site locations. A discussion about telemedicine can offer a viable alternative to outreach facilities if such methods exist in your program. This can be an appealing feature, as there can be an increase in work efficiency for certain contexts. If there are research interests from a faculty member, the funding sources, available startup funds, and protected time should be clearly stated.

### Compensation and incentives

Having clear incentive structures will function to both attract and retain new talent. The salary structure should clearly articulate whether this is a straight salary, salary plus bonus, equal shares, pay-for-performance, productivity wRVU based compensation, or a blended model. New physicians want to know how their salary would be at risk for extenuating circumstances such as reduced patient volume, disability, childcare, or illness. If joining a private practice, there should be a discussion about partnership, and what the buy-in requirements would involve. Additionally, the IR team needs to discuss the conditions for a new physician to be eligible for other financial incentives that involve payments from call coverage, committee work, or other non-clinical activities. Other non-salary benefits could also include disability protection, signing bonus, malpractice coverage, CME (Continuing Medical Education) credits, relocation reimbursement, and debt repayment.

Exploring Non salary quality of life benefits may also overvalue standard financial offerings. Avoiding seven-day weekly call coverage can be desirable even in low volume settings. For junior attendings, having mentorship options available help them feel more secure when consultation is available for patient and career decisions. Selling the setting of the practice can appeal to physicians with families where the cost of living, real estate, education and recreational opportunities, ease of commute, or popular family career options. Lastly, offering vacation scheduling for major holidays and ease of scheduling can mean that physicians can derive more value out of the vacation days that are provided.

### Career opportunities

IR physicians seek positions where there is support for career growth. In academia, having discussions with the team and department heads about mentorship and professional development should be established early in the physician's new position. Clear expectations about joining committees, additional training, and certifications. Furthermore, offering time allocation and infrastructure support by the team will help launch new investigative projects and yield more productivity from your team. Private practice physician groups must clearly delineate the requirements for that physician to earn partnership rights.

### Facilities and infrastructure

It is important that physicians feel that they have sufficient equipment and facilities to practice. Present the supporting equipment and facilities that your practice has available, including the basics discussed in this paper and any additional unique assets, such as office procedure rooms, hybrid OR equipment, fluoroscopy suites, and extensive endovascular equipment inventory. This data is valuable to provide, as the physician will need to know if the resources available will support his desired procedural workload. If the department shares space with other specialties, anesthesia is short staffed, or the time can be difficult to schedule, it may be challenging to promote junior attendings to join where limited resources will be disbursed based on seniority. Similarly, the quality of life can be negatively impacted for the IR physician if available block times are limited to Friday afternoons or weekends.

Personnel allocated for both administrative and clinical capacities should be available for ancillary support for new physicians. Given program variability, stating the presence of scribes, IT support, electronic medical record system and training, and coding responsibilities can provide an edge for your program's recruitment efforts. For value-based reimbursement systems, quality support for reimbursement will also impact income or administrative workload for physicians.

### Lifestyle

Promoting a healthy lifestyle can benefit the happiness of junior physicians while also benefiting the IR practice. Offering IR suites only at unpopular times, a disproportionate call allocation, poor schedule flexibility, and limited vacation time will have an impact on that individual's moral and team synergy. If they are highly desired by the new team member, the practice can leverage other financial incentives that will reduce expenses. The physician will be happier and perform better with the team.

### Inclusive hiring practices

The advertising and position details should be designed to be inclusive and appealing to all qualified applicants. IR has historically been a male dominated specialty, and a 2015 UK census has shown female labor force participation around 10% (240). The commonly cited concerns about focusing on IR were work/life balance, risks of radiation exposure, effect of pregnancy on training, and the male-dominated work environment (241). Advertising efforts to promote the position should make efforts to address what is being done in these areas of concern such that any concerned party can be aware how these obstacles can be overcome. Some non-financial benefits that improve quality of life can also attract unique qualified applicants that have other personal obligations that could be better balanced with a more flexible schedule.

### Environment

The cultural environment impacts team performance and ability to recruit and retain physicians. Poor morale can develop due to deficits in any listed domain. The hostile attitude will infect the expanded team including nurses, advanced practitioners, technicians, and trainees. Dedicated efforts must be made to audit the individuals and factors that corrupt overall morale because it will significantly hinder current performance, ability to grow the staff, and influence of the practice. Outside the department, greater hospital incentives such as collaboration facilitate IR ability to add value to patient care. If specialties are protective of their patients, it will be challenging to maintain growing patient flow. Lastly, having a positive active community with the public helps the physician feel fulfilled. Highlighting how new members of the team can enjoy the city culture can ensure that time spent outside of patient care makes them appreciate their work.

### IR marketing

**Figure 2:**
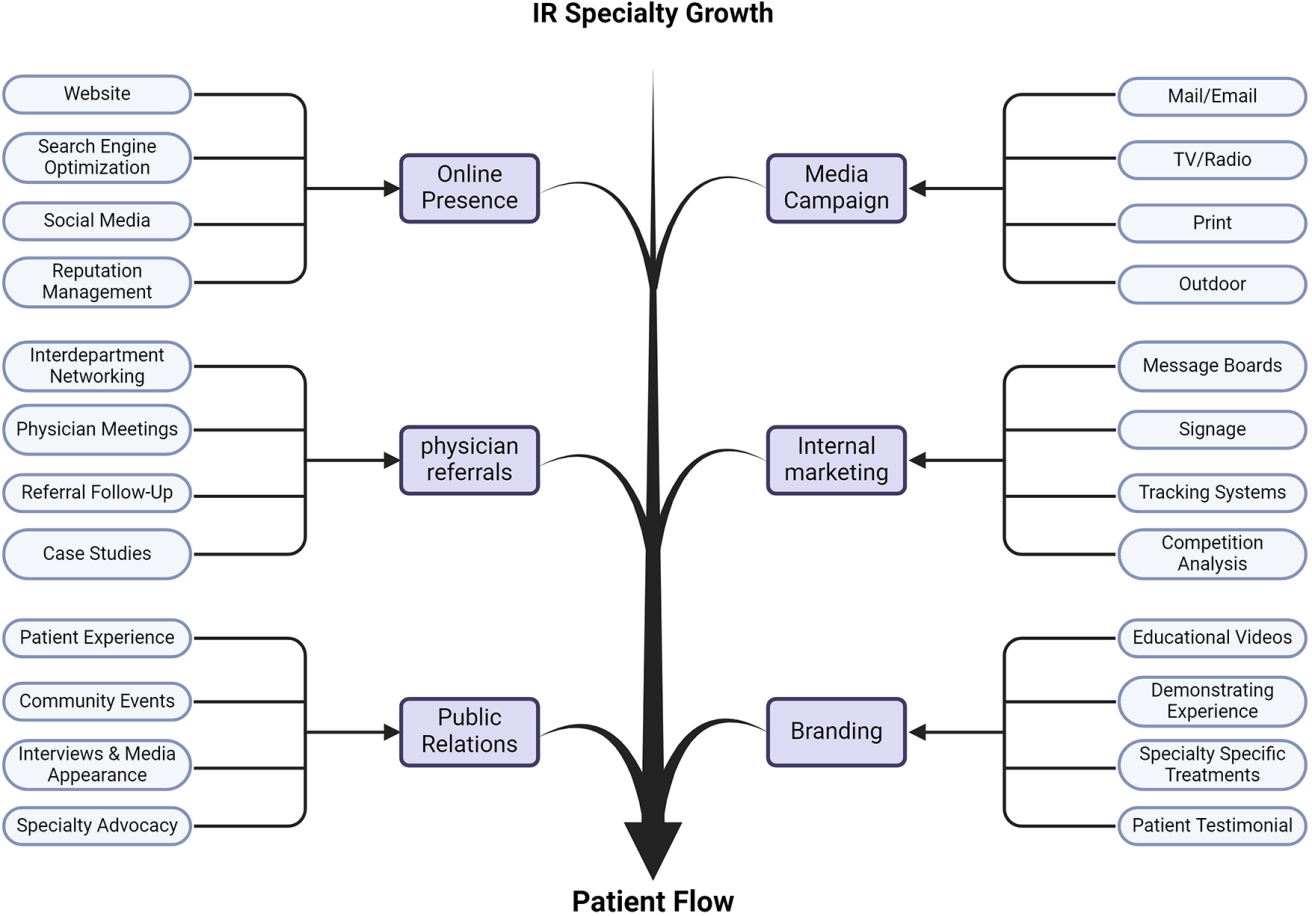
Expanding the value of IR. Figure outlines the various dimensions IR physicians and practices should address to grow the influence and importance of IR in the medical system. Approaches range from maximizing your visibility, to general societal and cultural branding. These dedicated foci require active participation to be successful and drive more patient flow through IR to benefit from IR innovation.

Like other businesses, there needs to be a concerted effort to get patients through your practice to grow your influence and provide work and revenue for your physician team (Figure 2). However, there are unique methodologies to growing an IR practice that are less draining on finances compared to paid advertising (242). First, in promotional opportunities be sure to promote the whole hospital system and the suite of programs that is offered. Overall awareness and recognition may lead to future referrals. If patients learn about your hospital system and utilize bariatric surgery, they might be good candidates for bariatric embolization, vertebroplasty from joint degeneration, or candidates for fibroid embolization. Similarly, oncological referrals can be high yield opportunities to intervene and help this population, as broadening the awareness of treatment options will make them more apt for Y-90 referrals. Experience has shown that utilizing your available network of physicians is an effective marketing option.

**Figure 3:**
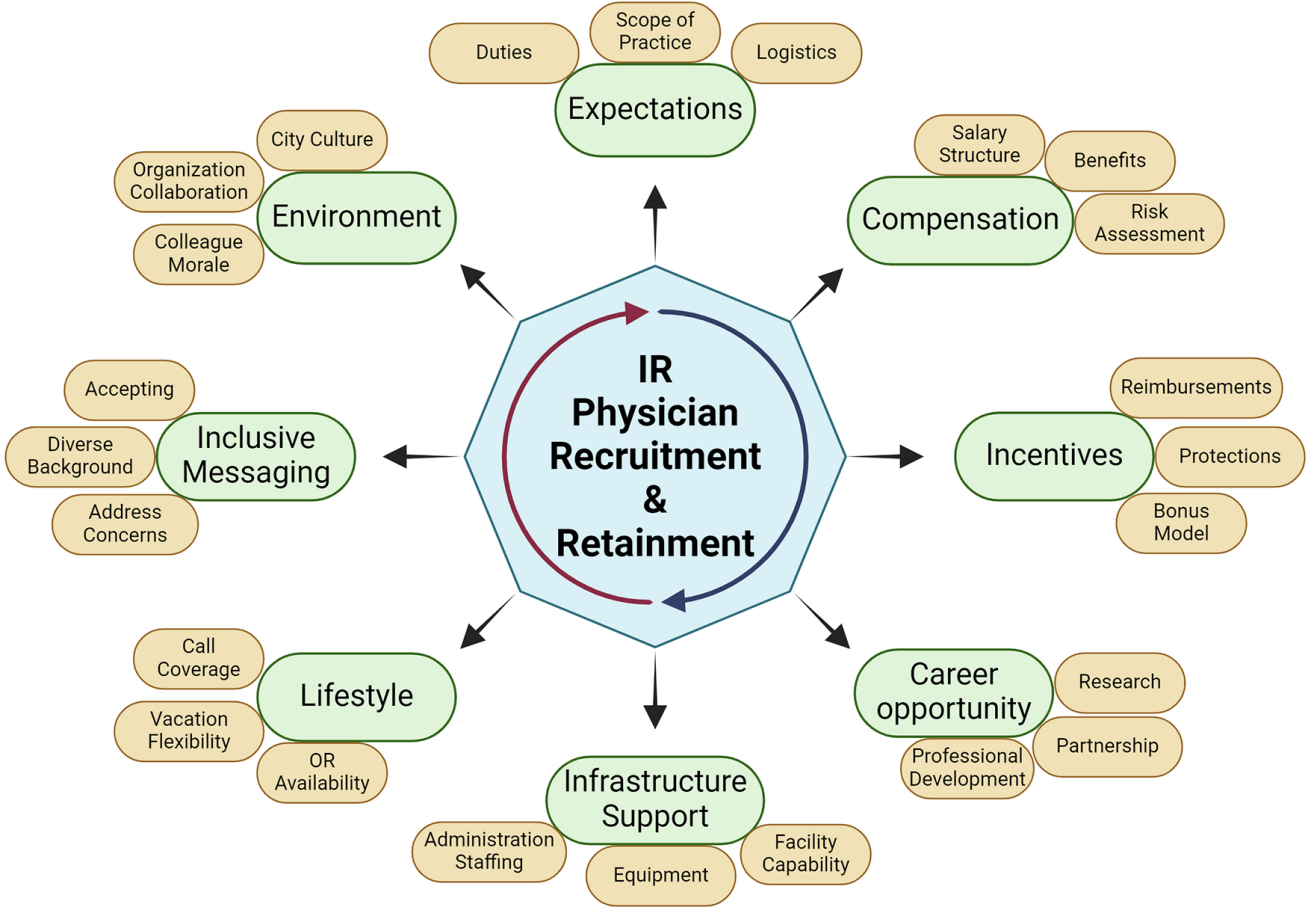
Model of IR physician recruitment and retainment. Each domain addresses an essential element in growing an IR practice that can operate successfully in the modern healthcare system. These elements begin with clear communication (expectations), followed by individual needs (compensation, incentives, career opportunity), followed by sociocultural desires (support, lifestyle, messaging, environment). Teams must periodically re-address these domains to accommodate change. Efforts in these areas ensure the team can focus on their collective mission of helping patients.

To best utilize marketing efforts, there are a few essential components (242). Having dedicated physicians who dedicate time to promote the program and the treatment opportunities to physicians and other public outreach. Have specific staff with marketing expertise to gain more visibility including social media, advertisements in traditional and alternative media, search engine optimization, email promotion, online presence, and networking management. Not only does this gain more visibility but also drives the prestige of your practice. Once all this work is focused on generating attention, internal validation should be done so that points of contact facilitate patient follow-up. Make sure that public lines have staff capable of answering questions regarding the staff, procedures, scheduling, and benefits that can be provided.

## Expanding the impact of IR

### Promotion and awareness

There are recent efforts in the field of IR research and development to expand the methodology for minimally invasive procedures. Importantly, IR needs to expand its development of new efforts to expand the visibility and influence of the field on medicine and the public (243–245). More efforts have been directed toward academia trying to influence the medical curriculum to broaden the exposure to IR in clinical rotation (246). This ensures that training programs for integrated and early specialization are filled and can expand new spots for talented new physicians. Dedicated efforts to spread the amazing work of IR should also expand to popular media, as the name “interventionalist” doesn't carry the same perceptual connotation as “surgeon.” Despite several high-profile cases, such as Melania Trump and Steve Scalise, there is little public recognition of IR contribution (247). While IR should reserve the use of “surgeon” for physicians who are board certified in surgery for descriptive accuracy, there is still an unresolved general branding issue.

### Governmental policy and healthcare infrastructure

Governmental advocacy and lobbying for pro-IR positions ensure the financial security of the field. If IR can justify its RVUs and still provide sufficient patient throughput to be profitable, hospital administrations will network and allocate resources to provide them with a stable source of revenue. Similarly, because of the challenges of other specialties evolving expertise in minimally invasive and endovascular procedures, sufficient funding from the National institutes of Health. Professional societies focus on these fronts, but they are small compared to other specialties. By increasing our value to all the stakeholders in the modern medical landscape, the synergistic effects of common goals will lead to a strong future for IR physicians and new developments to drive more efficient patient care.

### Future directions

The types of minimally invasive procedures continue to expand as modern technology improves current techniques. The fundamental principle of remaining a valuable specialty is developing new methodologies to improve patient outcomes while reducing morbidity and mortality. The inception of the field was based on integrating innovative ideas into practice. Some of these include advances in robotics and artificial intelligence (AI). Robotics can assist in a wide array of procedures from improving the speed, accuracy, and radiation exposure of localized tumor ablation. Alternatively, robotic systems can improve intravascular wire navigation with similar advantages (1).

AI's impact will broadly impact the practice of medicine, including IR. AI tools can streamline clinical practice and reduce inefficiencies in workflow from scheduling, consenting, or monitoring patient messages. AI can provide direct medical assistance in the rapidly improving capability to communicate scientific literature, evaluate patient pre-procedural imaging, as well as complex procedural recommendations (2). While this technology is still prone to significant errors limiting its role in clinical decision making, AI will only continue to improve. Physicians should be open to exploring these tools to maximize the advantages IR practice and benefit to patients.

## Conclusions

IR provides value for diverse needs within the medical system. Patients are the primary focus and beneficiary of the techniques and innovations to medicine. IR developments offer new alternative procedures that demonstrate reduced cost, recovery times, and fewer complications relative to historical medical and surgical therapies. Hospitals and healthcare payers benefit by achieving similar outcomes more efficiently. Regardless of how the reimbursement system is structured, IR is a diverse medical specialty involving diagnostics and procedures capable of providing financial value. While benefits are gained on the patient level, collective efforts must be invested into expanding IR's market capitalization. This starts with optimizing a personal practice with the right team and equipment. The expanded growth of IR is accomplished with a focus on recruiting, networking, marketing, and public advocacy. IR has a responsibility to foster leaders in the healthcare space to support the mission to revolutionize how medicine is practiced (248, 249).
